# The Tolerogenic Function of Regulatory T Cells in Pregnancy and Cancer

**DOI:** 10.3389/fimmu.2019.00911

**Published:** 2019-05-08

**Authors:** Nanna Jørgensen, Gry Persson, Thomas Vauvert F. Hviid

**Affiliations:** Department of Clinical Biochemistry, Centre for Immune Regulation and Reproductive Immunology (CIRRI), The ReproHealth Consortium ZUH, Zealand University Hospital, and Department of Clinical Medicine, University of Copenhagen, Copenhagen, Denmark

**Keywords:** regulatory T cells, immune tolerance, cancer, immunotherapy, pregnancy, preeclampsia, HLA class Ib

## Abstract

Regulatory T cells, a subpopulation of suppressive T cells, are potent mediators of self-tolerance and essential for the suppression of triggered immune responses. The immune modulating capacity of these cells play a major role in both transplantation, autoimmune disease, allergy, cancer and pregnancy. During pregnancy, low numbers of regulatory T cells are associated with pregnancy failure and pregnancy complications such as pre-eclampsia. On the other hand, in cancer, low numbers of immunosuppressive T cells are correlated with better prognosis. Hence, maternal immune tolerance toward the fetus during pregnancy and the escape from host immunosurveillance by cancer seem to be based on similar immunological mechanisms being highly dependent on the balance between immune activation and suppression. As regulatory T cells hold a crucial role in several biological processes, they may also be promising subjects for therapeutic use. Especially in the field of cancer, cell therapy and checkpoint inhibitors have demonstrated that immune-based therapies have a very promising potential in treatment of human malignancies. However, these therapies are often accompanied by adverse autoimmune side effects. Therefore, expanding the knowledge to recognize the complexities of immune regulation pathways shared across different immunological scenarios is extremely important in order to improve and develop new strategies for immune-based therapy. The intent of this review is to highlight the functional characteristics of regulatory T cells in the context of mechanisms of immune regulation in pregnancy and cancer, and how manipulation of these mechanisms potentially may improve therapeutic options.

## Introduction

**Figure 1:**
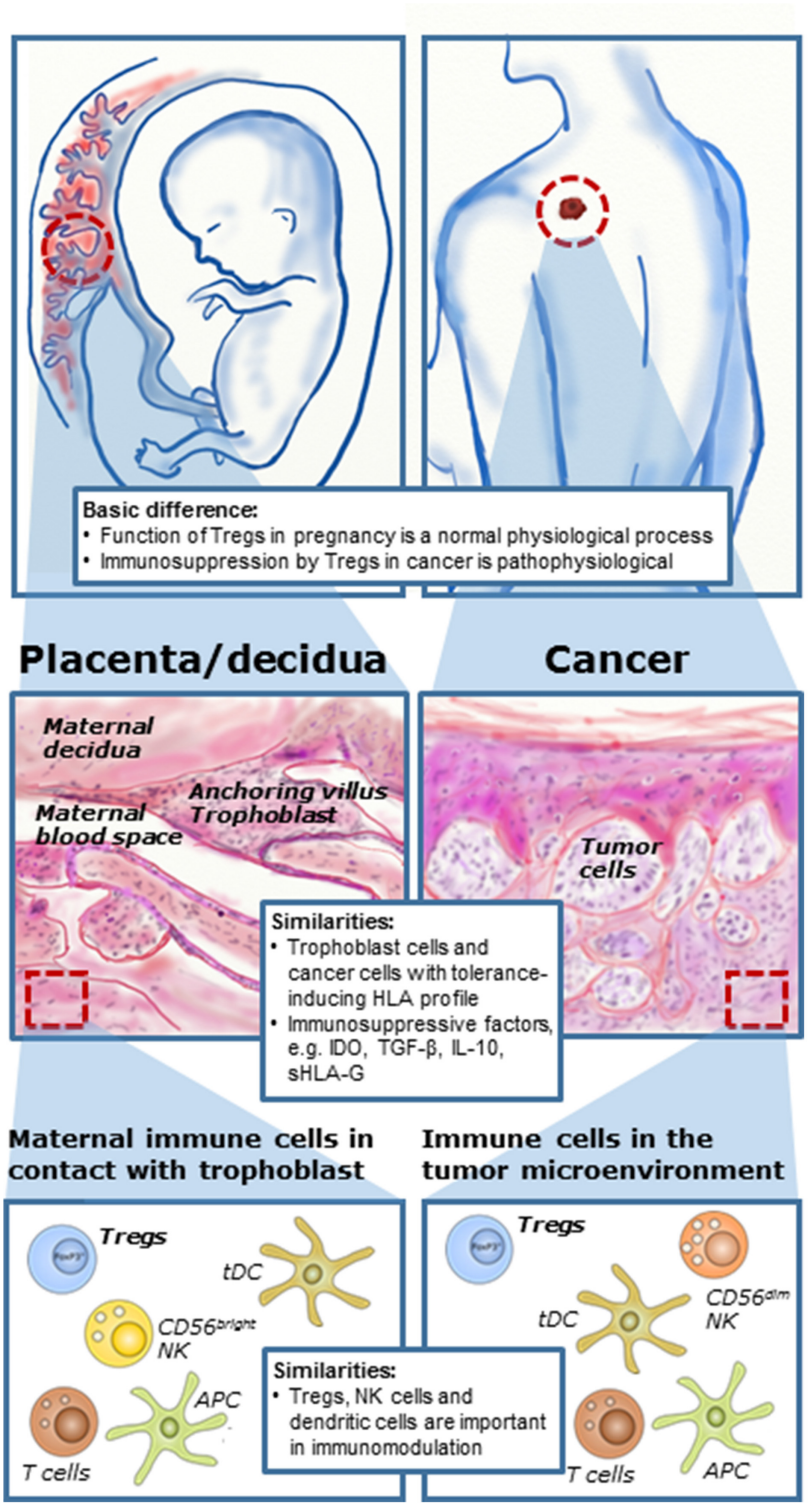
Immune mechanisms during pregnancy and cancer development. Although immunomodulation during pregnancy is a physiological process and in cases of cancer a pathophysiological process, there are a number of similarities in cellular and molecular mechanisms at the feto-maternal interface and in the tumor microenvironment. Tumors and fetuses seem to exploit some of the same immunomodulating mechanisms. Formation of the placenta during pregnancy involves invasion of fetal trophoblast cells into the maternal tissue for anchoring and vascular adaptions. In cancer, local invasion into neighboring tissue is essential for manifestation of malignant growth and the first stage in development of secondary tumors or metastases. Furthermore, several immune cells are present both at the feto-maternal interface and in the tumor microenvironment, here with malignant melanoma as an example. There is increasing evidence that regulatory T cells play important roles both in cancer and in reproduction. [Illustration partly inspired by Holtan et al. (5)].

Regulatory T cells (Tregs) constitute a dynamic and diverse T cell population composed of several subsets distinguished by phenotypic and functional characteristics. With their immunosuppressive properties, Tregs are central to the maintenance of immune homeostasis. They are implicated in critical immunoregulatory functions in several physiological conditions such as inflammatory responses, tissue repair, and reproduction. Furthermore, Tregs also play an important role in the pathophysiological immune tolerance induced by tumors (1–4). Hence, selective immunological tolerance is essential during any of these processes, and the mechanisms by which immune tolerance is sustained by Tregs might be similar. Some of the mechanisms responsible for induction of maternal immune tolerance during pregnancy may be the same as those involved in controlling an inflammatory response from not exaggerating beyond control, and furthermore the same mechanisms that may provide a pro-tumorigenic environment which allows cancer development. The role of Tregs is somewhat opposing in relation to a role in protecting the body and preventing disease development. Tregs must allow protective immune responses against pathogens and tumors, but simultaneously prevent inflammatory diseases by restraining aberrant responses to self and innocuous antigens with pregnancy as a borderline condition, where Tregs contribute to the establishment of active immune tolerance toward the fetus (Figure 1).

The similarities between reproductive biology and cancer development in terms of immunology is not that implausible. During pregnancy, the formation of the placenta involves the invasion of the semi-allogeneic fetal trophoblast cells into the maternal tissue for anchoring and vascular adaptions, such as formation of spiral arteries providing nutritional support for the growing fetus. The maternal immune system has to allow this invasion of partly foreign cells to ensure a successful pregnancy. Thus, cancer cells and cells of the developing placenta both share the capacity to invade normal tissue and create a microenvironment that support immunologic privilege and angiogenesis (Figure 1). The proliferation and migration of cancer cells at a distant site mediated in part by modulation of a tolerogenic immune response in the tumor microenvironment may be compared to the situation in pregnancy, in which the developing placenta invades the uterus and a semi-allogenic fetus escapes rejection from the maternal immune system (5–7). A prominent hypothesis states that the failure to establish immune tolerance during pregnancy may lead to pregnancy complications or pregnancy loss. However, this may indicate that it should be possible to exploit the same mechanisms responsible for immune regulation during pregnancy in treatment of cancer and to reject cancer cells by immunological mechanisms (5). Finally, it is important to remember that immunomodulation and immunosuppression during pregnancy are physiological mechanisms but in cases of cancer they are pathological and in most cases unfavorable.

The function of Tregs as potent anti-inflammatory cells has led to considerable interest in their therapeutic potential. In cancer, there has been much progress within the field of immunotherapy within the last decade. Especially, cancer therapy by inhibition of negative immune regulation is already used in the clinic. Manipulation and propagation of Tregs and their therapeutic application is a promising approach in order to reach a clinical benefit for affected patients (8–10).

As briefly mentioned above, while pregnancy is a physiological process in which the presence of Treg cells is favorable, cancer is a pathophysiological scenario in which the suppression of a potential anti-tumor response is undesirable. However, as will be discussed in later sections, this distinction is not always obvious, and in some cancer settings, the presence of Treg cells and thus the control of the inflammatory environment can probably be advantageous seen from an anti-tumor perspective. This highlights the importance of broadening our understanding of the function of Treg cells across different physiological and pathophysiological settings, such as pregnancy, pregnancy complications, and cancer, in order to develop and offer the right therapeutic treatment. This review provides an overview of current knowledge on the tolerogenic function of Tregs in immunological mechanisms during pregnancy and cancer, and in relation to possible therapeutic intervention of both human malignancies and reproduction.

## Regulatory T Cells

Regulatory T cells are a T lymphocyte population with immune suppressive properties responsible for maintaining antigen-specific T cell tolerance. Tregs comprise both CD4^+^ and CD8^+^ subtypes. Whereas, CD4^+^ Treg cells have been extensively studied, lack of clear markers to distinguish CD8^+^ Tregs from conventional CD8^+^ T cells has led to unsatisfactory characterization of origin, function and phenotype (11, 12). Therefore, this review will focus mainly on CD4^+^ regulatory T cell subsets, and “Treg” or “regulatory T cell” will refer to CD4^+^ regulatory T cells, unless stated otherwise.

Normally, CD4^+^ Tregs constitute 5–10% of the total CD4^+^ T cell population and are derived from thymic precursors (13). Regulatory T cells where first described in 1972, where Gershon et al. showed that T cells were capable of suppressing the antigen-induced response of other T cells directly without the mediation of B cells and their production of antibodies (14). However, it was not until 1995 that Tregs were identified as a specialized CD4^+^ T cell population expressing CD25 (15). Subsequently, several *in vitro* studies showed that CD4^+^CD25^+^ T cells represent a distinct lineage of naturally anergic and suppressive cells (16, 17). The original studies on characterization of Tregs were performed in mice. However, in 2001 a T cell population with identical immunosuppressive properties was identified in humans (18–21). In 2003, the transcription factor forkhead box protein P3 (FoxP3) was identified as a potent marker for Tregs in several mouse studies. FoxP3 deficiency caused a fatal lymphoproliferative disease demonstrating that the transcription factor was essential for development of Tregs and for their immunosuppressive function (22–24). The requirement of FoxP3 expression for immunosuppression was later demonstrated in humans (25).

Based on these discoveries, expression of CD25 on the cell surface and presence of the intracellular transcription factor FoxP3 became the key characteristics of the Treg population. The mutual expression of these markers is commonly used for identification of Tregs in experimental settings. Conversely, some studies suggest a lack of correlation between CD25 and FoxP3 in human and mice CD4^+^ T cells (24, 26). Alternatively, Liu et al. found that low expression of CD127 serves as a good biomarker for human Tregs together with CD25 expression (26), although other studies have not been able to find a clear correlation between CD127^lo^ and FoxP3 expression (27). In addition, several sub-populations of CD4^+^CD25^−^FoxP3^−^ Tregs have also been identified (28). Hence, the most specific marker still remains a matter of debate. Nevertheless, as expression of FoxP3 has been shown to correlate with suppressor activity irrespectively of CD25 expression many consider FoxP3 as the most specific Treg marker (29).

### Regulatory T Cell Subsets

**Figure 2:**
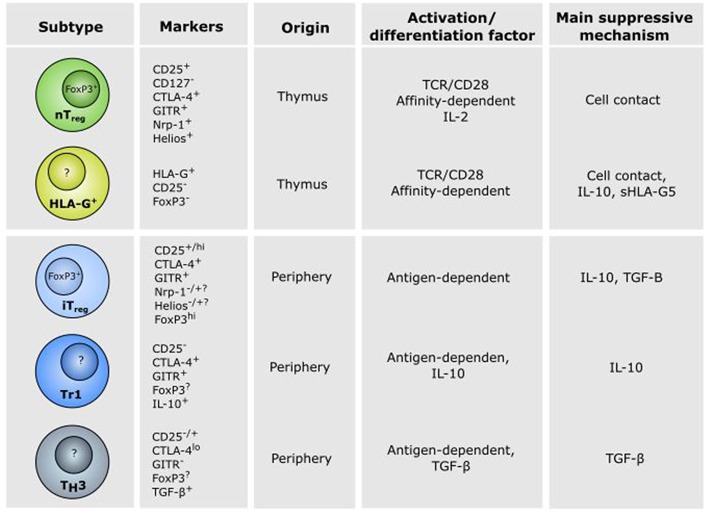
Characteristics of CD4^+^ regulatory T cell subsets. Different subsets of CD4^+^ regulatory T (Treg) cells exist and play a role in the establishment of tolerance in different physiological and pathophysiological settings. Thymic (t)Tregs and HLA-G^+^ Tregs are developed in the thymus in response to self-antigen, whereas induced (i)Tregs, Tr1 cells and Th3 cells are developed in the periphery in response to antigen presentation and cytokines. Natural Treg and iTregs are characterized by CD25 and FoxP3 expression, while HLA-G^+^ Tregs, Tr1, and Th3 cells are CD25^−^FoxP3^−^, although controversies do exist (see the text for details). The thymus-derived Treg cells mediate their effect mainly through cell contact. In contrast, immune suppression by peripheral induced iTreg, Tr1, and Th3 cells are mediated mainly via secretion of the anti-inflammatory cytokines TGF-β and IL-10.

Tregs are found throughout the body, where they modulate activities of cellular components of both the innate and adaptive immune system. CD4^+^ Tregs can be divided into distinct subsets according to unique functional and homeostatic properties (Figure 2). FoxP3^+^ Tregs originating from the thymus, where they have differentiated during T cell ontogenesis, are referred to as natural or thymic (t) Tregs, and Tregs developed in the periphery or *in vitro* from conventional CD4^+^ T cells are referred to as peripheral or induced (i) Tregs (30, 31). Furthermore, there are two phenotypically distinct immunosuppressive subtypes of the iTregs, namely the IL-10 producing T regulatory type 1 (Tr1) cells and the TGF-β-producing Th3 cells (32, 33). It remains to be determined, whether the different subsets of Tregs belong to unique cell lineages, or whether they only reflect the plasticity of the Treg population and represent an altered state of differentiation (34). Furthermore, it is debated, whether iTregs can arise from any conventional T cell or from a pre-committed cell lineage (35).

**Figure 3:**
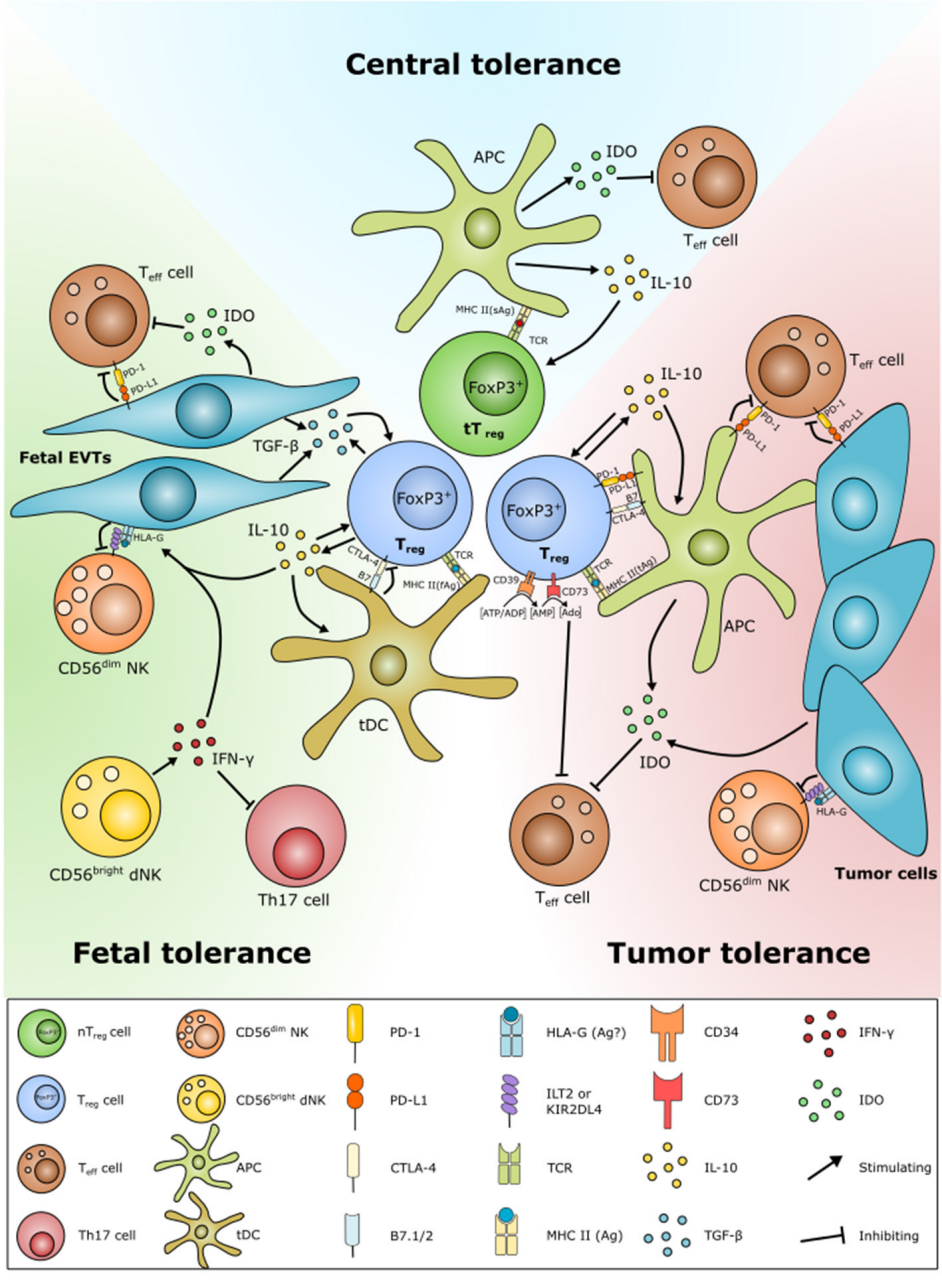
Schematic overview of similarities in Treg function in central tolerance, fetal tolerance, and cancer tolerance. Tolerance play an important role in both fetal and cancer tolerance. Tregs are developed by presentation of antigens of fetal (fAg) or tumor (tAg) origin. Many tumor cells and fetal extravillous trophoblast (EVT) cells have both diminished or no expression of MHC class II and classical MHC class I molecules. Instead, the EVT cells and some cancer cells express HLA class Ib molecules, e.g., the immune modulatory non-classical HLA-G. HLA-G is able to protect fetal and tumor cells from NK cell lysis, as well as according to a few studies to induce Treg formation. Fetal EVTs and tumor cells are also able to contribute to Treg homeostasis by inhibiting effector T cell activation and proliferation through PD-L1/PD-1 and indoleamine-2,3-dioxygenase (IDO) expression. Decidual (d)NK cells further contribute by inhibiting Th17 responses by IFN-γ expression. Fetal EVTs also express cytokines, e.g., IL-10 and TGF-β that induce Treg development. Tregs limit Teff cells and promote their own proliferation and survival through direct engagement with Teff cells, e.g., via PD-L1/PD-1, by the conversion of ATP to Adenosine (Ado) and cytokine secretion.

Both tymus-derived tTregs and peripheral iTregs are characterized by high expression of CD25, FoxP3, cytotoxic T-lymphocyte-associated protein 4 (CTLA-4), and glucocorticoid-induced tumor necrosis factor-related receptor (GITR), but iTregs have been shown to express reduced levels of programmed cell death protein 1 (PD-1), CD73, the transcription factor Helios and the surface antigen neutropilin-1 (Nrp1) (36). Both Helios and Nrp1 have been suggested as markers for distinguishing between tTregs and iTregs, but the specificity of these markers is a current matter of debate (36–39). Mice studies have suggested that GITR is involved in the generation and maturation of FoxP3^+^ tTregs and Tr1-like cells (40, 41). Furthermore, it has been suggested that GITR is a marker of active Tregs (42). In addition to the above mentioned markers, expression of the ATP-degrading enzymes CD39 and CD73 on the surface of Tregs have been increasingly used as markers of Tregs and might contribute to the suppressive activity together with expression of the immunoglobulin-like transmembrane protein LAG3 (Figure 3) (43–46).

Thymic CD4^+^CD25^+^ tTregs are developed in the thymus from CD4^+^ precursors. Development of tTregs or conventional CD4^+^ T cell populations from the CD4^+^ precursor depends on the affinity of the T cell receptor (TCR) for self-antigens: low affinity leads to positive selection of conventional CD4^+^ T cells, whereas medium affinity interactions with thymic epithelial cells lead to development of CD4^+^CD25^+^ tTregs (47–49). Immunosuppression by tTregs require activation via their TCR. When activated, the suppressor effector function is independent of antigen-specificity. Conversely, inhibition of the effector T (Teff) cell population is mainly depending on cell contact and independent of suppressive cytokines (18, 50). The result of tTreg mediated immune regulation is reduced number of Teff cells and altered activity and trafficking pattern of activated Teff cells (37).

*In vitro* or *in vivo* induced iTregs can be differentiated from naïve CD4^+^ T cells in response to antigen, CD28, TGF-β and IL-2 stimulation, and mediate their suppressive activity mainly via secretion of cytokines such as IL-10 and TGF-β that reduce the capacity of dendritic cells (DCs) to present antigen (37). As for the iTregs, the peripheral Tr1 and Th3 subsets are also induced in the periphery from the conventional CD4^+^ T cells. In contrast to tTregs and iTregs, expression of CD25 and FoxP3 in Tr1 and Th3 cells are controversial (51–53). Tr1 and Th3 have been identified as FoxP3- and CD25-negative, although it seems that expression of both markers can be upregulated in response to activation (53, 54). The Tr1 cells were first described by Groux et al. (55), who found that Tr1 cells are activated by IL-10 and suppress the proliferation of CD4^+^ cells in response to antigen (55). Presence of IFN-α further enhances IL-10-mediated induction of Tr1 activation and differentiation (56). The Tr1 cells constitute a low proliferating subset that produces high levels of IL-10, low levels of TGF-β and marginal or no IL-2 and IL-4 (55, 57). Th3 cells are activated upon antigen stimulation (58). However, TGF-β also promote the induction of Th3 cells from CD4^+^ T cells, which can be further enhanced by the presence of IL-10 and IL-4 (32). When active, the Th3 cells have suppressive properties for Th1 and Th2 cells through secretion of TGF-β (59, 60).

A new subset of regulatory T cells have emerged during the recent years defined by expression of the immunosuppressive Human Leukocyte Antigen (HLA) molecule HLA-G (Figure 2). In 2007, Feger et al. identified HLA-G^+^ T cells among CD4 and CD8 single-positive cells in the peripheral blood and thymus from healthy individuals (61). The cell population showed reduced proliferation to allogeneic and polyclonal stimuli and the suppressive effect of HLA-G expression was confirmed by neutralization of HLA-G on CD4^+^HLA-G^+^ cells, which reduced their suppressive capacity. The cells were, however, not expressing CD25 and FoxP3, like previously described Tregs. When comparing the properties and molecular characteristics of CD4^+^HLA-G^+^ cells and CD4^+^CD25^+^FoxP3^+^ cells, there is a clear distinction between the phenotype and the cytokine profile of the two cell populations (62). The suppressive function of CD4^+^HLA-G^+^ cells is mediated mainly by secretion of soluble HLA-G and high levels of IL-10 and IL-35, while CD4^+^CD25^+^FoxP3^+^ cells seems to work mainly in a cell-contact dependent manner. Furthermore, CD4^+^HLA-G^+^ cells are clearly distinct from Tr1 cells as they do not require the presence of other cell types (63). The identification of a novel T cell population with regulatory properties expressing HLA-G on the surface has led to the notion of a new subset belonging to the repertoire of suppressor T cells (64). As these cells have a similar function as CD4^+^CD25^+^FoxP3^+^ Tregs, their role in peripheral immune regulation is increasingly recognized. However, whether they should be identified as traditional regulatory T cells as the classical Tregs is somehow controversial.

No universal agreement on which factors that can be used to differentiate tTregs from iTregs seems to exist. Moreover, it is important to note that most studies have used shared markers such as FoxP3, CD25, and CD127 for identification of Treg cells, thus do not differentiate between tTregs and iTregs, and by that definition also exclude any immunosuppressive FoxP3^−^ T cells, such as the Tr1, Th3, and HLA-G^+^ Tregs. The following section will focus on studies using FoxP3, CD25, and CD127, and the term “Treg” will therefore refer to regulatory CD4^+^ T cells regardless of origin, unless specifically stated otherwise.

## The Role of Tregs in Cancer

The progression of cancer is controlled by a complex biologic system that is highly dependent on interaction between the malignant cells and the surrounding tumor microenvironment comprising the immune cells. Various types of immune cells can infiltrate the tumor and may influence tumors differently depending on their histological and molecular type, their stage, the microenvironment of the organ in which they occur, and the nature of the tumor (Figure 1) (65). Immune effector cells can detect and destroy cancer cells preventing tumor formation by means of both the innate and adaptive immune compartments. However, the anti-tumor activity of the immune cells are often downregulated by tumor-derived signals leading to immune escape. A large number of preclinical models have demonstrated the influence of Tregs in development and progression of several types of malignancies, and Tregs are generally believed to be a significant contributor to tumor immune escape (66, 67). A widely accepted hypothesis is that recruitment of tumor-infiltrating Tregs with immunosuppressive properties enable the malignant cells to evade the host immune response (68).

Increased numbers of tumor-infiltrating Tregs have been demonstrated in patients with ovarian (69), liver (70), melanoma (68), gastric, and esophageal cancer (71), in chronic lymphocytic leukemia (72), and in breast cancer, where it is associated with a more aggressive phenotype (73). The same is seen in gastric and esophageal cancers, where Tregs increase with disease stage suggesting induced expansion of Tregs by tumor-related factors (74). Furthermore, Treg numbers are also increased in the peripheral blood of patients with breast, pancreatic (75), liver (70), gastric, and esophageal cancer (71) in comparison with blood from healthy individuals.

Various studies have tried to identify the role of Tregs in immune evasion, and as it has become clear that the effect of Tregs on tumor progression vary according to the tumor type, the prognostic significance of Treg infiltration remains a matter of debate. An overview of the clinical significance in a range of cancers is provided in Table 1. In connection to the role of Tregs in evading immune recognition, a common presumption is that high numbers of Tregs within lymphoid infiltrates can be predictive of relapse and dead. However, the prognostic value of Tregs is somehow controversial as in some cancers Tregs infiltration may exert a beneficial role or can have both a negative and positive effect on disease progression and survival. The negative effect on survival is observed in pancreatic (87), liver (90), gastric, and esophageal cancer (74). It is though more likely to observe opposing roles of Tregs in terms of survival in a wide range of cancer types such as cases of ovarian carcinoma (69, 76), colorectal cancer (85, 86), melanoma (88), breast cancer (77–84), head and neck squamous cell carcinoma (91, 92), and lymphoma (93–96), where a high frequency of Tregs improve disease-specific survival in some patients and in others favors immune escape and tumor growth. Furthermore, in some patients there is no correlation between Treg infiltration and disease progression at all (89). The reason for this discrepancy in the prognostic value of Treg infiltration might be related to the different nature of the cancers and the effect of inflammation on tumor growth, but could also be dependent on the presence of different Treg subsets in the different malignancies.

**Table 1:** Examples of clinical significance of Tregs in the tumor microenvironment.

**References**	**Cancer**	**Presence of Tregs**	**Treg definition**	**Effect on clinical outcome**		**Comment**
				**Good/bad**	**How**	
Curiel et al. (69)	Ovarian carcinoma	High	CD4^+^CD25^+^FoxP3^+^	Bad	Reduced survival	Treg cells suppress tumor-specific T cell immunity and contribute to growth of human tumors *in vivo*
Milne et al. (76)	Ovarian carcinoma	High	FoxP3^+^	Good	Increased disease-specific survival	Tregs is associated with survival only in high-grade serous tumors from optimally debulked patients
Gobert et al. (77)	Breast cancer^a^	High	CD4^+^CD25^hi^ CD127^lo^FoxP3^+^	Bad	Higher risk of relapse and death	Tregs are selectively recruited within lymphoid infiltrates and activated by mature dendritic cells through tumor-associated antigens
Demir et al. (78)	Breast cancer^a^	High	FoxP3^+^	Bad	Shorter overall survival	The density of Treg infiltration before chemotherapy is a strong predictor for survival
Sun et al. (79)	Breast cancer^a^	High	FoxP3^+^	Bad	Shorter disease-free survival	Significant correlation between expression of PD-1 in tumor-associated immune cells and FoxP3^+^ cells
West et al. (80)	ER^−^ breast cancer	High	FoxP3^+^	Good	Prolonged recurrence-free survival	FoxP3^+^ Tregs are positively correlated with CD8^+^ cytotoxic T cells and anti-tumor immunity
Bates et al. (81)	ER^+^ breast cancer ER^−^ breast cancer	High	FoxP3^+^	Bad -	Shorter relapse-free and overall survival No impact	High Treg numbers associated with high-grade tumors and lymph node involvement
Liu et al. (82)	ER^+^ breast cancerER^−^ breast cancer	High	FoxP3^+^	Bad Good	Poor survival Improved survival	High FoxP3^+^ cell numbers are associated with young age, high grade, ER negativity, concurrent CD8^+^ T cell infiltration, and HER2 positive ER^−^ subtypes
Lee et al. (83)	Triple-negative breast cancer	High	CD4^+^CD25^+^FoxP3^+^	Good	Improved survival	High infiltration of FoxP3^+^ Tregs is an independent prognostic factor for overall survival and progression free survival
Liu et al. (84)	Triple-negative breast cancer	High	FoxP3^+^	Good	Better overall and disease-free survival	Elevated expression of Treg and immune-related genes is associated with more favorable outcome
Frey et al. (85)	Colorectal cancer	High	FoxP3^+^	Good	Improve disease-specific survival	High frequency of tumor-infiltrating Tregs is associated with early T stage, tumor location, and increased 5-year survival rate
Chang et al. (86)	Colorectal cancer	High	CD4^+^CD25^+^FoxP3^+^	Bad	Favor tumor growth	CCL5/CCR5 signaling recruits Tregs to tumors and enhance their ability to kill antitumor CD8^+^ T cells leading to immune escape
Kono et al. (74)	Gastric and esophageal cancer	High	CD4^+^CD25^hi^	Bad	Poor survival rate	After curative resections of gastric cancers, the proportion of Tregs is significantly reduced. In cases with recurrent tumors, levels increase again
Hiraoka et al. (87)	Pancreatic ductal adenocarcinoma	High	CD4^+^CD25^+^FoxP3^+^	Bad	Poor prognosis	The prevalence of Tregs increase significantly during the progression of premalignant lesions
Miracco et al. (88)	Primary cutaneous melanoma	High	CD4^+^CD25^+^FoxP3^+^	Bad	Predictive of recurrence	The percentage of Tregs, both among tumor cells, inside tumor parenchyma and at periphery, is significantly higher in cases that recurred
Ladányi et al. (89)	Primary cutaneous melanoma	High	FoxP3^+^	-	No prognostic impact	The degree of Treg infiltration do not correlate with tumor thickness, metastasis, or survival.
Kobayashi et al. (90)	Hepatocellular carcinoma	High	CD4^+^CD25^+^FoxP3^+^	Bad	Lower survival	The prevalence of Tregs increase in a stepwise manner during the progression of hepatocarcinogenesis
Badoual et al. (91)	Head and neck squamous cell carcinoma	High	CD4^+^FoxP3^+^	Good	Favorable	Regulatory CD4^+^FoxP3^+^ T cells are positively correlated with locoregional control
Drennan et al. (92)	Head and neck squamous cell carcinoma	High	CD4^+^CD25^inter/hi^ CD127^lo/−^	Bad	Favor tumor progression	Elevated frequency and suppressive activity of CD25^hi^ Tregs is associated with advanced tumor stage and metastasis to lymph nodes
Tzankov et al. (93)	Lymphomas	High	FoxP3^+^	Good	Improved survival	Increased number of FoxP3^+^ cells positively influence survival in follicular lymphoma, germinal center-like diffuse large B cell lymphoma, and Hodgkin's lymphoma
Carreras et al. (94)	Follicular lymphoma	High	FoxP3^+^	Good	Improved overall survival	Patients with low Treg numbers presented more frequently with refractory disease
Alvaro et al. (95)	Hodgkin's lymphoma	Low	FoxP3^+^	Bad	Unfavorable	Low infiltration of Tregs in conjunction with cytotoxic lymphocytes is predictive of unfavorable outcome
Schreck et al. (96)	Hodgkin's lymphoma	High	FoxP3^+^	Bad/–	Shorter disease-free survival	A high ratio of Treg over Th2 cells is associated with shortened disease-free survival. Tregs have no prognostic impact alone

a *Cohort of patients with advanced/invasive breast cancer irrespective of molecular subtype*.

### Mechanisms of Treg-Mediated Immunosuppression in Cancer

Several mechanisms contribute to the accumulation of Tregs within neoplastic lesions including increased infiltration, local expansion, survival advantage, and development from conventional CD4^+^ cells (30). All of these mediated through signaling with other cells and through different signaling molecules (Figure 3).

Studies on mice deficient of key markers of Tregs, including IL-10, CTLA-4, GITR, or PD-1 that develop severe immune-related disorders indicate that these molecules are crucial for Treg function in a cancer setting. The CTLA-4 receptor is a negative regulator of T cell responses functioning as an immune checkpoint. Leach et al. was the first to show that blockades of the inhibitory signals of CTLA-4 enhance antitumor immunity in mice (97). Proof that this was also the case in humans came in 2003 in a clinical investigation, where CTLA-4 blockade increased tumor immunity in some previously vaccinated melanoma and ovarian carcinoma patients (98). Much research have been performed on the mechanism of antitumor immunity elicited by CTLA-4 blockade and one study has shown that Treg-specific CTLA-4 deficiency results in downregulation of CD80 and CD86 on DCs leading to loss of immunosuppression (99). This happens in part through abrogated expression of the immunosuppressive enzyme indoleamine 2,3-dioxygenase (IDO) by the DCs (100). When it comes to cancer, IDO is expressed also within solid tumors from both tumor and stromal cells, where it under normal conditions restrains an inflammatory reaction against cancer cells by degradation of tryptophan and recruitment of Tregs (101, 102). Another commonly known checkpoint molecule is PD-1. PD-1 is a receptor expressed on activated T cells, B cells, and myeloid cells. One of the early proofs of PD-1 being involved in maintenance of self-tolerance came in 1999, where a knock-out mouse model showed that a defect in the PD-1 gene specifically predisposes to development of lupus-like autoimmune disease suggesting that PD-1 serves as a negative regulator of immune responses (103). The same was seen in humans, where a study by Freeman et al. revealed that engagement of PD-1 by its ligand PD-L1 led to inhibition of T cell receptor-mediated lymphocyte proliferation and cytokine secretion (104). Furthermore, blockade of PD-1 seems to enhance recruitment of Teff cells in intrasplenic tumors and prevent metastatic spread of several different cancers (105). The crucial role of CTLA-4 and PD-1 in regulation of a tolerogenic immune response opens up for a blockage of both checkpoint molecules that may have great therapeutic potential in terms of activating an immune response against the cancer cells. Whereas, both CTLA-4 and PD-1 function as negative regulators, GITR function as a co-stimulatory receptor, leading to activation, proliferation and cytokine production in both Teff and Treg cell populations (106–108). As mentioned, GITR is expressed in high levels by Tregs, and has been shown to be increased in several cancer forms including breast cancer (42, 109, 110). Engagement of GITR on Treg cells has been shown to inhibit their suppressive function, and rendering Teff unresponsive to Treg-mediated suppression (106, 107). However, it has also been shown that GITR induces IL-10 production, that if blocked leads to further GITR-mediated proliferation (108), leaving the exact role of GITR controversial. As shown in a study, the function of GITR on Treg cells is most likely context-dependent and rely on the model used to study its function, as well as the immunological milieu (111). Nevertheless, GITR is, like CTLA-4 and PD-1, an attractive target for immunotherapy, and GITR triggering using agonist antibodies and Fc-GITRL abrogates Treg-mediated suppression (106).

Whereas, the function of tTregs is mainly cell-cell contact-dependent, the secretion of soluble factors, such as cytokines by iTregs and other Treg subtypes is essential for their function (Figure 2). IL-10 is a cytokine produced by CD44^hi^ Tregs and plays a central role both in parasitic infections (112), intestinal inflammation (113), and cancer (114) again emphasizing the involvement of similar mechanisms in different pathophysiological conditions. In addition to IL-10, TGF-β is also produced by peripheral Tregs. Both IL-10 and TGF-β have pleiotrophic functions and have been implicated in both cancer progression as well as clearance [reviewed by (115)]. The effect of IL-10 and TGF-β therefore most likely depends on the specific cancer type, and therapy targeting these cytokines should be done with careful considerations. Considering that Treg cells are defined as CD25^hi^, the high affinity IL-2Rα chain, IL-2 is another cytokine central to both thymic and peripheral Treg development, function and homeostasis (116, 117). In contrast, the IL-7 receptor α chain, CD127, is low or absent in human Tregs, indicating that IL-7 is not required for Treg function, although a study in mice has suggested that IL-7 might be involved in early Treg development and in development of CD4^+^FoxP3^lo^ Tregs (116, 117).

Another increasingly acknowledged mechanism involved in development of cancer is regulation of the expression of HLA molecules in the tumor microenvironment. Increasing evidence suggest that expression of the classical and non-classical HLA class Ia (HLA-A, HLA-B, HLA-C) and class Ib (HLA-E, HLA-F, HLA-G) molecules influence immune regulation in a coordinated action with Tregs. This influence of HLA molecules is seen in multiple physiological and pathophysiological processes as the antigen-presenting capability of HLA molecules play a crucial role in infectious diseases, graft rejection, autoimmunity, reproduction, and cancer. Deregulation of the HLA class I molecules on the cancer cells leads to evasion of the host immune system (118). In a recent study on the prognostic value of tumor-stroma ratio combined with the immune status of the tumor, Vangangelt et al. showed that breast cancer patients with a stroma-low tumor and expression of classical HLA class I molecules have a better prognosis compared to patients with a stroma-high tumor and downregulation of HLA class I (119). Furthermore, when expression of HLA class Ia molecules are concomitantly lost, high expression of HLA-G is associated with a worse relapse-free period in breast cancer (120) and is suggested to facilitate invasion and increase the metastatic capacity of invasive ductal breast carcinoma (121–123). In gastric cancer, HLA-G expression significantly correlates with the presence of Tregs and is predictive of poorer survival (124). Expression of HLA-G and the presence of FoxP3^+^ tumor-infiltrating lymphocytes is also believed to contribute to the suppression of effective T cell immune responses in melanoma (68, 125). We have recently shown an association between high HLA-G expression and a high frequency of FoxP3^+^ tumor-infiltrating lymphocytes in malignant melanoma patients (126). Furthermore, in an *in vitro* study we have demonstrated that the HLA-G^+^ choriocarcinoma cell line JEG-3 originating from placenta upregulates Tregs, and that the level of pro-inflammatory cytokines is modulated through HLA-G (127).

A subset of HLA-G-expressing T cells have also been shown to play a role in promoting a tolerogenic tumor microenvironment. A recent study found a population of CD4^lo^HLA-G^+^ T cells associated with development of castration-resistant prostate cancer in prostate cancer patients after treatment with androgen deprivation therapy. Expansion of the CD4^lo^HLA-G^+^ cells resulted in impaired immune surveillance and a tumor microenvironment that were permissive of tumor growth (128). In pregnancy, CD4^+^HLA-G^+^ T cells have been reported and may be reduced in pre-eclampsia, although knowledge of a possible role of this subset is currently very limited (129).

We are currently investigating how expression of HLA class Ia and Ib expression modulate the immune response in breast cancer with emphasis on Tregs and Natural Killer (NK) cells. By studying molecular and genetic changes of the immune cells in contact with tumor cells we aim to identify molecular markers associated with the regulatory function of the immune cells and clinical outcome. Identification of regulatory immune cell gene signatures in tumors can be important and relevant when assessing the clinical course of the disease and prognosis. A recent study focusing on immunogenic gene signatures in triple-negative breast cancer found a high expression of tumor-infiltrating lymphocyte gene signatures in the tumor compared to normal tissues and that elevated levels of Treg gene sets were consistently associated with better overall survival and disease free survival (84). This confirms the controversy about the prognostic significance of Tregs in the tumor microenvironment and emphasizes the importance of research that can elaborate on the role of Tregs in a specific cancer setting and for the individual patient.

Substantial redundancy may exist in the mechanisms essential for establishment and maintenance of immune tolerance (46). Hence more research is necessary to identify mechanisms that could constitute the best targets for immunotherapeutic treatment strategies.

### Antigen-Specific Tregs

With the aim to elucidate the role of Tregs in cancer development, several studies have found that the Treg response is an early event preceding the activation of Teff cells (130, 131). It was seen many years ago in mice, that a regulatory immune response are present early followed by a decrease in cellular reactivity against the tumor cells and a progressive loss of immune recognition correlated with progression of tumor growth (132). A mechanism by which Tregs are stimulated by the presence of the tumor is via recognition of antigens.

Tumors are believed to present tumor-specific antigen in the form of neo-epitopes, sometimes known as tumor-associated antigens. Since tumor cells originate from normal cells and develop within the context of self-tissue, most cancer antigens are self-antigens, and the immune mechanisms that prevent immune recognition of the tumor cells might function in similar ways as those that prevent autoimmune attack of normal tissue (133, 134). This is contrary to pregnancy, where both foreign and self-antigens are present from the semi-allogenic fetus and immune suppression is necessary in order to avoid fetal rejection. However, it may be emphasized that cancer cells might eventually also, due to high mutational rate, express antigens foreign to the body that can be recognized by Teff cells.

A previous study by Wang et al. characterized tumor-specific CD4^+^ T cells derived from a melanoma patient and were the first to isolate antigen-specific Tregs, and further showed that cell-cell contact was required for T cell-mediated immune suppression in agreement with previous studies (135). The group identified Tregs specific for LAGE1 and afterwards the ARCT-1 peptide (136). Tregs specific for a broad range of tumor antigens including melanoma tissue differentiation antigens and cancer-testis antigen, have been identified in patients with metastatic melanoma (137), and following studies performed in colorectal cancer have also revealed tumor antigen-specific Tregs (138). In colorectal cancer patients undergoing resection, a high level of FoxP3^+^ Tregs specific for tumor antigens drives immunosuppression and correlates with tumor recurrence and relapse (139). Studies in diabetic mice have revealed a superior immunosuppressive activity for antigen-specific Tregs compared to non-specific Tregs (140, 141). Furthermore, Tregs responding to self-antigens have also been shown to suppress anti-tumor immune responses (142, 143). Indications are that Tregs are likely to play an important role in cancer immunology and elaborating on the specificity of Tregs involved in antitumor responses could be beneficial from a therapeutic perspective.

### Immunotherapeutic Intervention in Cancer

Given the role of Tregs in immune evasion and tumor progression, several studies have already suggested that they are promising as therapeutic targets (31). Initially, studies focused almost exclusively on the cancer cells as targets for therapeutic interventions. However, cancer cells frequently acquire therapeutic resistance because of inherent genetic instability. Hence, working toward manipulation, propagation, and therapeutic application of Tregs will provide new and improved treatment options. The prognostic effect of Tregs in different cancer types is important to take into consideration when selecting a treatment strategy, and even though Tregs appear as an obvious target for anti-tumor treatment, manipulation of Treg mechanisms is not that simple and more selective approaches for therapeutic strategies are needed. This involves targeting of specific Treg subsets and the inhibition or activation of Tregs depending on the type of cancer (30). Furthermore, the composition of other immune cells in the tumor microenvironment must also be taken into account when assessing whether a patient will benefit from immunotherapy. Recently an immune biomarker task force elicited by the Society for Immunotherapy of Cancer (SITC) sought to make recommendations of immune-related biomarkers that can predict the outcome of immunotherapy in cancer patients (144). They focus on biomarkers in the tumor microenvironment, gene expression at the tumor site, tumor antigens, mutational load, peripheral biomarkers, and host-related genetic biomarkers. Overall, this suggest that a combination of personalized diagnostics is necessary in order to assess immunocompentence of the individual. In terms of this, an analysis of immune gene signatures should be feasible to determine the potential for immunotherapy. Liu et al. performed an extensive analysis on immunogenic signatures in triple-negative breast cancer on two large-scale breast cancer genomic datasets. They demonstrated that this type of breast cancer has a strong tumor immunogenicity, which suggested that these patients could benefit from immunotherapy (84).

Even though treatment by activation of the immune system have proved to be successful it is not without side effects. One of the biggest challenges of targeting Tregs and blocking immune checkpoints is the development of severe system immune-related side effects. Releasing the brake on the immune system can lead to a systemic immune activation and might cause extensive autoimmune reactions (31).

One branch of immunotherapy evolves around the idea of activating the immune system targeting the regulatory mechanisms that suppress an immune response against the cancer. Especially, cancer therapy by inhibition of negative immune regulation has proved very successful within recent years in the form of immune checkpoint inhibitors and are currently used in cancer immunotherapy. Discovery of the two checkpoint molecules CTLA-4 and PD-1 that function as brakes on the immune system has led to a new approach for treating cancer patients. Ipilimumab and tremelimumab are two well-characterized anti-CTLA-4 antibodies, the first approved for treatment of malignant melanoma, colorectal cancer, and renal cell carcinoma and the second being tested in clinical trials on colorectal cancer and lung cancer patients (145–151). Pembrolizumab is an anti-PD-1 drug approved for treatment of multiple cancers including cervical cancer and melanoma (152–155). Nivolumab is another anti-PD1 drug that in combination with ipilimumab is used as first-line treatment of melanoma being more effective than either agent alone (156). Furthermore, nivolumab is shown to have a higher efficacy as compared to chemotherapy in patients with melanoma, who progressed after CTLA-4 treatment (157). These immunotherapies have emphasized how manipulation of immune regulation is essential for eradicating tumors.

Another strategy of breaking the tolerance to tumor tissue is to inhibit the IDO pathway. Studies show that elimination of IDO-positive immunosuppressive cells change the regulatory microenvironment (158). Furthermore, it was found that 1-methyl-tryptophan isomers capable of blocking IDO activity is effective in reversing the suppression of T cells promoted by DCs (159). Combined with other immune activating drugs, IDO might also enhance the efficacy of immunotherapy by preventing counter-regulation in response to immune activation (160). Combining induction of IDO-specific immune responses with anti-cancer immune therapy has the synergistic potential to both eliminate cancer cells and immune suppressive cells expressing IDO (158). Hence, clinical trials have been initiated to evaluate the efficiency of IDO inhibitors and IDO-based vaccinations. A combination of pembrolizumab and the selective IDO inhibitor Epacadostat initially showed promising results increasing the anti-tumor activity in patients with advanced solid tumors in a phase I/II study (NCT02178722) (161). Unfortunately, no benefit in survival was observed with the combined treatment compared to pembrolizumab alone in the following phase III clinical trial (NCT02752074) (162). A clinical phase I study have shown that a vaccine with an epitope derived from IDO is well-tolerated in patients with metastatic non-small cell lung cancer (NCT01219348) (163). Currently, a clinical phase 2 study is testing a combination therapy of the PD1 antibody Nivolumab and a vaccine consisting of PD-L1 and IDO (NCT03047928).

A third way to enhance anti-tumor effects is to deplete Tregs in the tumor microenvironment. Mouse studies have proven the effectiveness of eliminating Tregs by administration of IFN-γ and the use of IL-2 antibodies in combination with stimulation of effector immune cells (140, 164). An ongoing clinical trial is investigating a combination of pembrolizumab and low-dose IL-2 in patients with advanced melanoma or renal cell cancer (NCT03111901). Furthermore, a phase I/II study have shown that CD4^+^CD25^+^ Treg depletion improves the graft-vs.-tumor therapeutic effect of donor lymphocyte infusion in patients suffering from hematopoietic malignancies and relapse after standard allogeneic hematopoietic stem cell transplantation (NCT00987987) (165).

Another branch of immunotherapy focuses on targeting tumor antigens. Recognizing an increased activity for Tregs that are antigen-specific gave the idea that Tregs could also be exploited to target cancer cells. Expression of chimeric antigen receptor (CAR) T cells to engineer T cells with antigen-specificity toward cancer cells have already offered a promising strategy to target diseases with extensive immune activation. This directs the attention to a similar approach for Tregs with the possibility that CAR Tregs could be used in Treg-mediated therapy reducing a generalized immunosuppression (35). In terms of this, studies have shown that it is possible to isolate CD4^+^CD25^+^ cells with immunosuppressive function from peripheral blood and expand them *in vitro* without loss of function, which represent a major advance toward the therapeutic use of these cells in T cell-mediated diseases (166). So far, engineered Tregs have been shown to target the central nervous system reducing symptoms of multiple sclerosis by suppression of inflammation and in colitis patients CAR T cells could hinder development of colorectal cancer (167, 168). This indicate that the use of engineered Tregs is preferred in cancers with prominent inflammation and where immune suppression will have a beneficial role in preventing tumor progression. Moreover, a new study suggest a promising role for CAR T cells in delivery of checkpoint inhibitors. Mouse CAR T cells was modified to secrete PD-1 blocking single-chain variable fragments and was shown to enhance the anti-tumor function in mouse models of hematologic and solid tumor (169). Hence, the targeted delivery of immune checkpoint inhibitors or expression of other immunomodulatory molecules could prevent systemic blockade, eventually improving treatment and minimizing adverse side effects.

## The Role of Tregs in Reproductive Biology

With the inheritance of half of the genes from the father, the fetus is considered to be semi-allogenic in an immunological sense. This results in the immunological paradox in which the maternal immune system has to be able to tolerate the presence of the foreign paternally derived antigens for a successful pregnancy to take place. Initially, a shift from a Th1 pro-inflammatory response toward an anti-inflammatory Th2 response has been the central paradigm to explain the generation of fetal tolerance (170). However, during normal pregnancy the decidua contains a decreased CD4^+^/CD8^+^ ratio compared to the peripheral blood, and decreased numbers of CCR6^+^ Th1, Th2, and Th17 cells, while CCR6^−^ Th1 cells and CD4^+^CD25^hi^FoxP3^+/hi^ Tregs are increased (171, 172). This reflects a much more complex scenario, and are now explained as a balance between Th1, Th2, Th17, and regulatory responses involving both innate and adaptive immune cells (173, 174). Moreover, recently it has been proposed that the immune system plays different roles in the different phases of pregnancy; an inflammatory response seems necessary for the implantation of the blastocyst, while there is an establishment of a tolerogenic milieu for maintenance of the pregnancy, and yet another shift toward inflammation at parturition (174, 175). To constrain inflammation and avoid fetal rejection, several mechanisms have developed in which increasing focus has been giving to the role and function of the anti-inflammatory properties of the regulatory Tregs (10, 173, 174, 176), which is described in the next section.

Although maternal Teff cells are fully capable of recognizing paternal antigens and become activated, this does not lead to rejection of the fetus (177, 178). Tafuri et al. were also able to show that paternally derived tumor cells were able to persist during pregnancy independent of antibody response, but was rejected after parturition (178). This indicates a pivotal role for establishment of a temporal state of tolerance against the paternal antigens during pregnancy, and thus an important role for Tregs (178, 179). Several mechanisms have been identified that protect the fetus from immune attack, including attenuated expression of polymorphic Major Histocompatibility Complex (MHC)/HLA proteins as well as expression of the nearly monomorphic HLA class Ib molecules, release of anti-inflammatory hormones, cytokines, and immunomodulatory molecules by the placenta, and suppression of allo-reactive responses (173). Fetal tolerance during pregnancy seems to be a balance between clonal exhaustion (i.e., deletion or inactivation) of allo-reactive T cells and immune regulation—a phenomenon also seen in transplantation (180–182).

During the formation of the maternal-fetal interphase fetal trophoblast cells will invade into the maternal decidua harboring maternal immune cells to form the placenta. In parallel, the tumor microenvironment can be seen as a pathological situation with tumor cells with a distinct and possible non-self-phenotype in close contact with immune cells (Figure 1). The placenta is regarded as an immunological privileged site and is the source of many immunomodulatory molecules, hormones and cytokines that contributes to establishment of fetal tolerance (183). Roughly speaking, there are two compartments in the placenta in which maternal immune cells interact with the fetal cells; the intervillous space and the decidua. The interactions between the fetal trophoblast cells and the maternal T cells will be different at the two places. The intervillous space is the space between the anchoring villi, flooded with maternal blood that allows exchange of nutrients. The syncytiotrophoblast cells here lack the expression of all MHC/HLA molecules and should, in theory, not be able to interact with the maternal T cells (184). It has been suggested that the main role of the T cells located here is to protect mother and fetus against infectious pathogens (185). However, it should be noted that maternal antigen presenting cells (APCs) are still able to induce an adaptive immune response by presenting paternal antigens despite the lack of MHC/HLA on the syncytiotrophoblast cells (186). In contrast, invading extravillous trophoblasts (EVTs) present in the decidua express a unique combination of HLA-C and the non-classical HLA-E, -F, and -G molecules, enabling them to elicit immunosuppression and induce tolerance. The expression of a polymorphic paternally inherited HLA-C molecule on EVT has the potential to induce alloreactivity toward the fetal-derived cells. However, HLA-C is only expressed at a level of ~10% of HLA-A and -B, and HLA-C interacts both with T cells and NK cells through KIRs (7, 184). In addition to the local immune changes happening in the placenta during pregnancy, peripheral tolerogenesis is also observed (187). It is not yet fully understood whether the peripheral changes reflects the local changes or if there is a separate systemic response to pregnancy, e.g., through interaction with shed trophoblast debris or exosomes.

Many studies have shown the importance of Tregs for pregnancy (10, 173, 174, 176). Tregs and FoxP3 mRNA have been found in the endometrium throughout the menstrual cycle, increasing in the follicular/estrus phase and thereby the receptive phase, suggesting that the uterus is preparing for pregnancy also involving immunomodulatory changes (188, 189). Some studies might also indicate that the female immune system is primed for pregnancy through contact with antigens and immunomodulatory molecules present in the seminal plasma during coitus (190). In mice, the CD4^+^ and CD8^+^ Treg populations expand immediately after mating due to activation by paternally derived antigens present in the seminal fluid (186). Pregnant women have a higher level of peripheral Tregs compared to non-pregnant women with Treg numbers peaking during first and second trimester (191–194). In parallel, higher levels of Tregs can also be observed in certain cancer patients compared with healthy individuals as discussed briefly previously. Moreover, it has been shown that women with infertility problems and women experiencing recurrent pregnancy loss (RPL) in first trimester have reduced number of Tregs and FoxP3 mRNA, indicating an early role for Tregs in the establishment of pregnancy (188, 195). The role of Tregs in connection with the uterine (u)NK cells in the endometrium of infertile women has been thoroughly described in a recent review by Kofod et al. (196). Reduced numbers of Tregs, and increased number of CD8^+^ T cells and Th17 cells, have also been associated with pregnancy complications such as pre-eclampsia (PE) and RPL (191, 192, 197). In mice, depletion of Tregs using anti-CD25 monoclonal antibodies at the time of implantation resulted in poor implantation and fetal reabsorption in allogeneic, but not in syngeneic pregnancies. In contrast, no effect was observed on either pregnancy outcome, blood pressure or urinary protein levels, when Tregs were depleted later in pregnancy (182). This confirms the proposed role for the Tregs in creating a tolerogenic environment toward the paternal allo-antigens early in implantation and pregnancy. It has been suggested that both thymic and induced peripheral Tregs play important roles in pregnancy. Mice studies have shown that pre-existing thymic memory/activated Tregs specific for self-antigens are present very early in pregnancy and thus play a role in implantation, whereas depletion of peripheral Tregs leads to increased abortion later in pregnancy (198). In human first trimester decidua, FoxP3^hi^ Tregs with a similar phenotype (CD45RO^+^HLA-DR^+^CTLA-4^+^) have been identified (171). Analysis of Treg cells from term placenta tissue also showed that these cells expressed GITR and had higher expression of CD25, CTLA-4, and CD69 in comparison to their peripheral counterparts, indicating an activated phenotype (194). Lastly, recent studies have shown that pregnancy also leads to the generation of both effector memory and central memory CD4^+^ and CD8^+^ T cells that persist after pregnancy (199). The development of memory Tregs after pregnancy and their possible role for subsequent pregnancies remains to be elucidated.

Despite these observations, the exact role of the Tregs are still poorly understood. Also, the activation and generation of Tregs are dependent on recognition of antigen. Although mice studies have shown that allo-reactive T cells are clonally deleted and inactivated in a paternal antigen-specific manner, and like-wise, that Tregs recognizing paternal antigens are generated during pregnancy, the exact nature and origin of the antigen responsible for generation of pregnancy-specific Tregs in natural settings are sparse (180, 182). More studies are needed to understand, whether the role of the Tregs is specifically to limit harmful pro-inflammatory/Th1 and allo-reactive immune responses toward the fetus, or whether the generation and function of the Tregs are to limit general inflammatory responses in an environment of tissue repair owed to the growing placenta (176).

### Mechanisms of Treg-Mediated Immunosuppression in Pregnancy

The mechanisms of fetal tolerance in pregnancy are many and cannot exclusively be attributed to the generation of fetal-specific Tregs. Tolerance include a balance between clonal deletion and/or inactivation of allo-reactive effector cells and immune suppression mediated by regulatory subsets comprising both innate cells, such as tolerance-inducing DCs, alternatively activated macrophages (M2) and the cytokine-producing CD56^bright^CD16^−^ decidual (d)NK cells, and adaptive cells, including CD4^+^ and CD8^+^ Tregs as well as regulatory B cells. All working together in an impressive network that secures a successful pregnancy (200–202).

Cells of the endometrium and placenta release numerous chemokines that play a role in orchestration of immunomodulatory cells (203). In contrast to dNK cells, which can be generated from CD34^+^ hematopoietic precursors present in the human decidua (204, 205), Tregs seem to be recruited to the uterus during estrus and in early pregnancy by chemokines such as CCL1, CCL4, CCL17, and CCL22 (171, 189, 206). In the pregnant mouse, the chemokine receptor CCR5 recognizing CCL4 is expressed by 70% of the CD4^+^CD25^+^ Tregs, and interaction of CCR8 with CCL1 has been shown to enhance the immunosuppressive function of the Tregs by inducing FoxP3 expression and IL-10, TGF-β and Granzyme B production (189, 207).

The fetal trophoblast cells also express and release a number of immunomodulatory molecules that contribute to the Treg balance. Importantly, as seen in cancer and discussed above, the attenuated expression of polymorphic HLA molecules in addition to the expression of the non-classical HLA class Ib, which show very limited polymorphism, are believed to protect the fetal trophoblast cells from a direct cytotoxic response by maternal Teff and NK cells (7, 208–210). Moreover, interactions with HLA-G have been shown to induce the development of immunosuppressive CD4^+^ T cells and suppress APCs (211, 212). A special CD8αα^+^ Treg cell that specifically identifies Qa-1a (equivalent to the human MHC class Ib molecule HLA-E), has been found to control activated CD4^+^ T cells in mice (213). Furthermore, CD8αα^+^ cells have been shown to infiltrate the ovaries during ovulation. Although the origin and characterization of the nature of the CD8αα^+^ cell was unclear, the CD8αα ^+^ cells seemed to originate from the thymus and responded to the thymus-expressed chemokine (TECK), which is important for T cell development. Importantly, it was found that depletion of the CD8αα^+^ cells resulted in impaired fertility of the female mice, suggesting a role in the establishment of pregnancy (214). The role of CD8^+^ Tregs in pregnancy is unclear, however, it would be interesting to study if any similar cell populations are important for pregnancy in humans.

Negative regulators such as PD-L1 (215), the TNF family members FasL (CD95L or Apoptosis Antigen (APO)-1L) and tumor necrosis factor-related apoptosis inducing ligand (TRAIL; CD235/APO-2L) (216–218) and IDO (219, 220) are also expressed by the trophoblast cells. These molecules, as described in previous sections, contribute to T cell homeostasis by inducing apoptosis in allo-reactive Teff cells. Moreover, the trophoblast cells also secrete IL-10 and TGF-β that contribute to Treg recruitment and differentiation (221, 222), of which IL-10 also has been shown to upregulate HLA-G, thus further contributing to the Treg balance (223). As mentioned earlier, IL-10 and TGF-β play an equally important role during cancer development. However, in a cancer setting their pleiotrophic function imply a more unclear effect on the Treg balance depending on cancer type.

The function of Tregs during pregnancy mirror those occurring in the tumor microenvironment, in which Tregs regulate other immune cells present in the maternal-fetal interphase (Figures 1, 3). Tregs limit the effect of allogen-specific Teff cells by the expression of CD25, CTLA-4, and the PD-L1 pathway and the secretion of IL-10 and TGF-β that induce apoptosis and suppress cytotoxicity in recipient cells (171, 173, 176, 197). PD-L1 expression by Treg cells has been found to inhibit proliferation of CD4^+^CD25^−^ T cells and suppress expression of the pro-inflammatory cytokines IFN-γ and TNF-α (224). PD-1 expression on T cells seem to be increased in healthy pregnancy compared to non-pregnant women (225), while reduced levels of PD-1 and PD-L1 have been suggested to promote Th17 proliferation, thus causing the Treg/Th17 imbalance observed in PE (226). Consistent with this, mice studies have shown that blocking of PD-L1 results in lower numbers of Tregs and increased Teff and Th17 populations, as well as increased fetal resorption and reduced litter size (227, 228). Moreover, engagement with PD-L1 and secretion of TGF-β promote the development of Tregs by increasing FoxP3 expression, and reducing Teff cell development (227). The immunosuppressive function of the PD1 pathway seems to work by similar mechanisms in cancer and pregnancy, though with opposite effect in terms of prognosis. Whereas, inhibition of the pathway is desirable for activating the immune response against cancer cells, activation and high PD-L1 expression is important in terms of promoting a healthy pregnancy.

The DCs are central for activation and differentiation of T cells by presenting antigen and providing co-stimulatory signaling. Formation of the placenta in early pregnancy is associated with increased number of tolerogenic immature (i)DCs. These cells have been shown to produce increased levels of IL-10 and induce Treg formation during pregnancy (229–231). Further, Tregs have been shown to induce the formation of anti-inflammatory alternatively activated macrophages (M2), partly by IL-10 (232). Moreover, Tregs secrete heme oxygenase-1 (HO-1) that keep DCs in an immature state in which they secrete higher amounts of IL-10 that further induce the formation of Tregs (233). In turn, these cells secrete IDO and TGF-β and engage with the CTLA-4 receptor on Tregs that together impairs allogen-specific T cell activity and induce Treg formation, further affecting the Tregs/Teff balance (234, 235).

Uterine and decidual NK cells play important regulatory functions for the vascularization and formation of the placenta in early pregnancy (236, 237). Like Tregs, a balance between cytotoxic CD56^dim^ and regulatory CD56^bright^ NK cells seems important for a successful pregnancy. Pregnancy complications such as RPL and PE have also been linked to a reduced CD56^bright^/CD56^dim^ NK cell ratio (238, 239). Tregs might also be important in regulation of the dNK cell phenotype. It has been shown that Tregs reduce cytotoxicity of NK cells in an TGF-β-dependent fashion and inhibit the release of IL-15 from DCs that are important for the generation of dNK cells (240, 241). Likewise, TGF-β secreted from decidual stroma cells has been shown to change the peripheral CD56^dim^ toward a decidual-like CD56^bright^ NK cell phenotype (242). It is likely that TGF-β secreted from Tregs will have a similar effect on the NK cell phenotype. On the contrary, NK cells are also able to contribute to the Treg homeostasis by reducing Th17 cell responses through the production of IFN-γ and inducing CD25^+^FoxP3^+^ Treg development (235, 243).

Apart from the classical CD4^+^CD25^+/hi^FoxP3^+^ T cells described above, other types of Tregs have also been associated with pregnancy. As briefly addressed in previous sections recent studies have identified an HLA-G-expressing CD4^+^ T cell population with immunosuppressive functions. The HLA-G^+^ Tregs show an activated/memory phenotype (CD25^+^CD45RO^+^) as the classical Treg cells, but lack the expression of FoxP3 (129). The CD4^+^HLA-G^+^ T cells are found at increased levels in peripheral blood in pregnant women compared with non-pregnant women. Additionally, one study reported that the placenta was enriched in CD4^+^HLA-G^+^ T cells compared to the peripheral compartment, and cases of PE have been associated with reduced levels of the CD4^+^HLA-G^+^ T cell subpopulation in both the decidua and in peripheral blood, indicating an important role for pregnancy (129). As mentioned earlier, HLA-G-expressing T cells are also observed in the tumor microenvironment promoting a tolerogenic immune milieu, but as with other immunological mechanisms having the same effect during pregnancy and cancer development, a favorable effect is actually opposite in the two settings. Immune suppression by HLA-G is crucial in terms of a healthy pregnancy, but unwanted in a cancer setting where it promotes tumor growth.

Taken together, it has become increasingly clear that Tregs are an important player in the complex network of immune cells that secure a healthy pregnancy. Regulatory T cells are central regulators at the maternal-fetal interphase, as well as in induction of peripheral tolerance during pregnancy. However, it is also evident that the Tregs cannot stand alone. The Treg cells regulate and are regulated by a variety of cells and immune modulatory molecules. Their exact role and the precise mechanism by which they exert their immune regulation needs to be further elucidated.

### Immunotherapeutic Intervention in Pregnancy Complications

Clinical treatments based upon immunomodulating Treg function in cases of infertility, pregnancy loss and pregnancy complications have not yet been implemented in routine settings.

Regarding the use of Treg measurements as a diagnostic or prognostic marker, Winger and Reed have reported an interesting but small study of 54 pregnant women with a history of infertility and/or pregnancy loss (195). In a new pregnancy, 23 of the women experienced another pregnancy loss in the first trimester, and 31 women were still pregnant after 12 weeks of gestation. The percentage of CD4^+^CD25^+^FoxP3^+^ Tregs in peripheral blood was significantly higher in the still pregnant >12 gestational week compared with the pregnancy loss group at mean day 49.2 ± 36.1 of the pregnancy. Based on the results from this pilot study the authors propose that measurements of Tregs may serve as a biomarker for the assessment of risk of pregnancy loss in newly pregnant women. Clearly, larger studies are needed to validate this.

In a rat model of pregnancy loss induced by the administration of lipopolysaccharide (LPS) resulting in maternal inflammation Renaud et al. showed that pregnancy loss could be prevented by immunomodulation (244). This was either accomplished by administration of IL-10 or by blockade of TNF-α by a TNF-α inhibitor (Etanercept). As discussed previously, studies especially in mice have shown the importance of the presence of Tregs for a successful pregnancy. In one study by Heitmann et al. a targeted depletion of Tregs was performed using a transgenic mouse model (245). It was observed that embryo implantation in syngenic matings was defective after Treg depletion. However, it was possible to restore embryo implantation by the transfer of Tregs into the mating mice. It can be speculated that administration or induction of pregnancy-related Tregs resembling engineered T cells used in cancer treatment could rescue some unsuccessful pregnancies caused by abnormal Tregs function either by aberrant number of cells or a functional defect. There might also be therapeutic potential in blockage or the administration of specific cytokines or HLA class Ib molecules locally in the female reproductive tract. In theory, such immunomodulation might be able to affect numbers or functionality of regulatory T cell subsets beneficial for a successful pregnancy. However, this therapeutic area clearly needs more studies primarily to clarify the basic mechanisms upon which new therapeutic strategies may be based on.

A reason as to why treatment based on immune modulation is not extensively studied in terms of pregnancy complications compared to the field of cancer immunotherapy, might be that the focus on the cause of pregnancy complications such as PE has been directed toward several different factors besides immune regulation.

## Conclusions and Perspectives

Many similarities exist in the regulatory immune landscape of the tumor microenvironment and at the feto-maternal interface during pregnancy (Figure 1). While trophoblast cells possess both maternal and paternal antigens, cancer is also a kind of a chimera consisting of cells presenting both self and tumor-associated antigens. Furthermore, it seems that the role of Tregs in pregnancy and cancer, modulating the host response directed toward foreign antigens in the placenta and the tumor, respectively, may not be very different. Keeping this in mind, the immunosuppressive role of Tregs in pregnancy is a physiological process, while the inhibitory role of Tregs in cancer is pathophysiological, which nevertheless also makes the elaboration of immune modulating capacity in both cases even more appealing. The apparent role of Tregs in early tolerance induction is another issue also important in both cancer and pregnancy. The early Treg response to embryo implantation is similar to those in a cancer setting with Tregs being activated within the first days of implantation and tumor emergence, respectively (5, 198). Most essential in reproduction and cancer immunology is the similar mechanisms of escape from host immunosurveillance mediated by Tregs in combination with other immune cells and immune factors. Therefore, investigating mechanisms engaging Tregs and their regulation in apparently distant fields like pregnancy and cancer have close connections and could be highly beneficial (246). This would involve a better mapping of cytokine networks and e.g., interactions with HLA class Ib molecules in both situations.

Investigating the similarities in immunity through the different trimesters in pregnancy and in advanced malignancies has the potential to advance the knowledge of mechanisms involved in Treg function and eventually help to overcome the burden of long-term antigen exposure and immunologic exhaustion. Treatment strategies can be aimed at aspects such as invasion, angiogenesis, immune privilege, and malignant proliferation (5). We can take advantage of the knowledge from the two different fields of cancer and pregnancy complications and potentially use it to facilitate the search for novel treatment strategies in either of them.

Modification of the presence of Tregs and the function of these cells have been studied more extensively in relation to cancer then in the case of pregnancy complications, and treatment strategies targeting immunosuppressive pathways are already established for some cancers. However, more discoveries on Treg regulation is essential for the exploitation of these cells both in the field of cancer and reproductive immunology in order to improve immunotherapy and to help prevent pregnancy complications. Similar for both fields, future research in interactions of Tregs with other cells, molecules responsible for recruitment of Tregs into the maternal-fetal interface and tumor site, and intracellular pathways of regulatory signaling in Treg cells, will be highly valuable. Especially knowledge about the interactions of Tregs with other immune cells is needed to provide safe treatment and to reduce immune-related side effects (246).

## Author Contributions

NJ, GP, and TH participated in the design and draft of the manuscript. NJ is the main author of sections dealing with Tregs in cancer, while GP drafted sections regarding Tregs in reproductive immunology. TH was responsible for overall supervision and did the final proofreading of the draft. All authors have read and accepted the final version of the manuscript. The figures and the table included in the article are made by the authors (Figure 1: TH and GP, Figures 2, 3: GP, Table 1: NJ), and the figures and the table have not been published before.

### Conflict of Interest Statement

The authors declare that the research was conducted in the absence of any commercial or financial relationships that could be construed as a potential conflict of interest.
